# Supplementary material for: Ensuring sexual and reproductive healthcare services amidst a pandemic: Experiences from health workers in Lima, Peru
Source: PLOS Glob Public Health. 2024 Jun 28;4(6):e0003187. doi: 10.1371/journal.pgph.0003187 (PMC11213333; doi:10.1371/journal.pgph.0003187)
Supplement: Attachment — Submitted filename: response to reviewers.docx [file pgph.0003187.s001.docx]

Ref: PGPH-D-23-01179 ENSURING SEXUAL AND REPRODUCTIVE HEALTHCARE SERVICES AMIDST A PANDEMIC: EXPERIENCES FROM HEALTH WORKERS IN LIMA, PERU

We want to thank the reviewers for their comments, which undoubtedly have allowed us to improve our article. We have read the report carefully and addressed each of them.

- Grammar: We have reviewed the grammar and addressed all the errors pointed out by reviewers.
- Methodology: We have addressed the comments made by the reviewers.
- Results: As suggested, we moved the paragraph describing the situation of the health services to section 2.1.
- Discussion. We agree with the reviewer; we are not addressing the issue of women's rights. We are focusing on the health system's capacity to respond and health workers' responses.
- Abstract: We have addressed the comments made to the abstract.

Best

Camila Gianella
