# Supplementary material for: Ensuring sexual and reproductive healthcare services amidst a pandemic: Experiences from health workers in Lima, Peru
Source: PLOS Glob Public Health. 2024 Jun 28;4(6):e0003187. doi: 10.1371/journal.pgph.0003187 (PMC11213333; doi:10.1371/journal.pgph.0003187)
Supplement: Attachment — Submitted filename: Response to reviewers.docx [file pgph.0003187.s003.docx]

Laura Miniea Hoemeke, DrPH
Academic Editor
PLOS Global Public Health

Ref: PGPH-D-23-01179 ENSURING SEXUAL AND REPRODUCTIVE HEALTHCARE SERVICES AMIDST A PANDEMIC: EXPERIENCES FROM HEALTH WORKERS IN LIMA, PERU

Dear Dr. Miniea Hoemeke

Firstly, we would like to thank the reviewers for their comments, which undoubtedly have allowed us to improve our article. We have read the report carefully and addressed each of them.

Grammar: We have reviewed the grammar and addressed all the errors pointed out by reviewers.

Explanation regarding the situation of the Peruvian health system (precarity): We have added information regarding this (and references).

References: We have reviewed all the references

Sincerely

Camila Gianella
