# Supplementary material for: Ensuring sexual and reproductive healthcare services amidst a pandemic: Experiences from health workers in Lima, Peru
Source: PLOS Glob Public Health. 2024 Jun 28;4(6):e0003187. doi: 10.1371/journal.pgph.0003187 (PMC11213333; doi:10.1371/journal.pgph.0003187)
Supplement: Attachment — Submitted filename: Response to reviewers.docx [file pgph.0003187.s004.docx]

Marianne Clemence
Staff Editor
PLOS Global Public Health

Ref: PGPH-D-23-01179 ENSURING SEXUAL AND REPRODUCTIVE HEALTHCARE SERVICES AMIDST A PANDEMIC: EXPERIENCES FROM HEALTH WORKERS IN LIMA, PERU

Dear Marianne Clemence

We have read the report carefully and addressed each of the points made in our article, as detailed below.

- **Literature review**: Paragraph 2 includes a detailed description of it. We have made some changes to paragraph 1 to clarify our point about the scarce literature and introduce paragraph 2 better. We also have included a study made in Peru in the references that we did not include in the previous versions.
- **References**: We have reviewed all the references. We are using EndNotes, so references are generated automatically.
- **COREQ**: We have attached the COREQ checklist
- **Supporting Information Files**: We have added a list of the supporting information files that have been uploaded.

Sincerely

Camila Gianella
